# Grouper Interferon-Induced Transmembrane Protein 1 Inhibits Iridovirus and Nodavirus Replication by Regulating Virus Entry and Host Lipid Metabolism

**DOI:** 10.3389/fimmu.2021.636806

**Published:** 2021-03-09

**Authors:** Ya Zhang, Liqun Wang, Jiaying Zheng, Liwei Huang, Shaowen Wang, Xiaohong Huang, Qiwei Qin, Youhua Huang

**Affiliations:** ^1^Joint Laboratory of Guangdong Province and Hong Kong Region on Marine Bioresource Conservation and Exploitation, College of Marine Sciences, South China Agricultural University, Guangzhou, China; ^2^Guangdong Laboratory for Lingnan Modern Agriculture, Guangzhou, China; ^3^Laboratory for Marine Biology and Biotechnology, Qingdao National Laboratory for Marine Science and Technology, Qingdao, China

**Keywords:** IFITM1, grouper, SGIV, RGNNV, viral entry, lipid metabolism

## Abstract

Interferon-induced transmembrane proteins (IFITMs) are novel viral restriction factors which inhibit numerous virus infections by impeding viral entry into target cells. To investigate the roles of IFITMs during fish virus infection, we cloned and characterized an IFITM1 homolog from orange spotted grouper (*Epinephelus coioides*) (EcIFITM1) in this study. EcIFITM1 encodes a 131-amino-acid polypeptide, which shares 64 and 43% identity with *Seriola dumerili* and *Homo sapiens*, respectively. The multiple sequence alignment showed that EcIFITM1 contained five domains, including NTD (aa 1–45), IMD (aa 46–67), CIL (aa 68–93), TMD (aa 94–119), and CTD (aa 120–131). *In vitro*, the level of EcIFITM1 mRNA expression was significantly up-regulated in response to Singapore grouper iridovirus (SGIV), or red-spotted grouper nervous necrosis virus (RGNNV) infection. EcIFITM1 encoded a cytoplasmic protein, which was partly colocalized with early endosomes, late endosomes, and lysosomes. The ectopic expression of EcIFITM1 significantly inhibited the replication of SGIV or RGNNV, which was demonstrated by the reduced virus production, as well as the levels of viral gene transcription and protein expression. In contrast, knockdown of EcIFITM1 using small interfering RNAs (siRNAs) promoted the replication of both viruses. Notably, EcIFITM1 exerted its antiviral activity in the step of viral entry into the host cells. Furthermore, the results of non-targeted lipometabolomics showed that EcIFITM1 overexpression induced lipid metabolism remodeling *in vitro*. All of the detected ceramides were significantly increased following EcIFITM1 overexpression, suggesting that EcIFITM1 may suppress SGIV entry by regulating the level of ceramide in the lysosomal system. In addition, EcIFITM1 overexpression positively regulated both interferon-related molecules and ceramide synthesis-related genes. Taken together, our results demonstrated that EcIFITM1 exerted a bi-functional role, including immune regulation and lipid metabolism in response to fish virus infections.

## Introduction

Interferon-stimulated genes (ISGs) are induced by interferon (IFN) through a series of signal transduction cascades, and exert virous antiviral effects at specific stages of the virus life cycle (e.g., inhibiting viral entry, gene transcription, and protein synthesis, assembly, and release) (1–3). IFN-induced transmembrane proteins (IFITMs) are one of the earliest identified ISG families, and play a crucial role in virus infection. IFITMs exert antiviral activity against a variety of RNA viruses, including influenza A virus (IAV) (4, 5), dengue virus (DENV), hepatitis C virus (HCV) (4, 6, 7), Ebolavirus (EBOV) (8), severe acute respiratory syndrome coronavirus (SARS-CoV) (9), human immunodeficiency virus 1 (HIV-1) (10), vesicular stomatitis virus (VSV), Scophthalmus maximus rhabdovirus (SMRV) (11, 12), respiratory syncytial virus (RSV) (13), Rift Valley fever virus (RVFV) (14), and Semliki Forest virus (SFV) (15, 16). Recently, IFITMs are also found to restrict some DNA viruses, including pseudorabies virus (PRV) (17), Rana grylio virus (RGV) (12), and vaccinia virus (VACV) (18). Studies on the mechanism of their antiviral actions demonstrated that IFITMs either restrict virus entry by suppressing viral fusion with the endosomal or lysosomal membrane (9, 19–22), or inhibit viral infection by regulating viral protein expression or interacting with viral proteins (23, 24). To date, five IFITM genes (IFITM1, IFITM2, IFITM3, IFITM5, and IFITM10) have been identified in humans, and three of them (IFITM1, IFITM2, and IFITM3) have been shown to function as restriction factors against different viruses. However, the function of other members still remains largely unknown (25, 26).

Singapore grouper iridovirus (SGIV) and red spotted grouper nervous necrosis virus (RGNNV) are important viral pathogens of groupers (*Epinephelus* spp.), a commercial cultured fish species in China and Southeast Asian countries (27, 28). SGIV was a highly pathogenic virus which was first isolated from the spleen of diseased grouper (*Epinephelus tauvina*), belonging to the genus *Ranavirus*, family *Iridoviridae* (27, 29). SGIV is an enveloped, large cytoplasmic DNA virus, and its genome is composed of 140,131 bp (30). RGNNV, a non-enveloped RNA virus, belongs to the genus *Betanodavirus*, family *Nodavirdae* and its genome mainly consists of two single-stranded positive-sense RNAs, including RNA1 (3.1 kb) and RNA2 (1.4 kb) (31). To clarify the host immune defense response against these two viruses (32, 33), numerous immune genes involved in virus infection have been characterized, such as IFN regulatory factor (IRF) 3 (34), IRF7 (35), mitochondrial antiviral signaling protein (MAVS) (36), stimulator of interferon genes (STING) (37), and cholesterol 25-hydroxylase (CH25H) (38). Recently, we found that grouper IFITM3 restricted viral entry to suppress iridovirus or nodavirus infectivity (39). Whether other IFITM members exerted crucial roles during grouper virus infection is worthy of investigation.

In this study, an IFITM1 homolog from orange spotted grouper (EcIFITM1) was cloned and characterized. The subcellular localization of EcIFITM1 was observed and its antiviral roles against both fish DNA and RNA viruses were investigated. In addition, the effects of EcIFITM1 on lipid metabolism and the host interferon response were determined *in vitro*. Our findings provided new evidence that EcIFITM1 played a bi-functional role in fish virus infection, including immune regulation and lipid metabolism.

## Materials and Methods

### Cells and Viruses

The grouper spleen (GS) and grouper brain (GB) cells which derived from the spleen and brain tissues of *E. akaara*, respectively, were cultured at 28°C in Leibovitz's L15 medium (Gibco, USA) containing with 10% fetal bovine serum (FBS; Gibco, USA) (40). SGIV and RGNNV stocks were isolated in our laboratory, and propagated in GS cells or GB cells, respectively (27, 41). Virus stocks were maintained at −80°C until experimental use.

### Gene Cloning of EcIFITM1 and Sequence Analysis

Based on the expressed sequence tag (EST) sequences of EcIFITM1 from the grouper spleen transcriptome (32), the open reading frame (ORF) of EcIFITM1 was cloned by PCR amplification. Next, the 5′ and 3′ ends of EcIFITM1 sequences were obtained using a SMARTer® RACE 5′/3′ Kit (Clontech, TaKaRa, Japan) and the primers were listed in Table 1. Using the BLAST program (http://www.ncbi.nlm.nih.gov/blast), we carried out the sequence analysis of EcIFITM1. In addition, the conserved domains were predicted using InterPro (http://www.ebi.ac.uk/interpro). Multiple amino acid (aa) sequence alignments were created using ClustalX1.83 software and edited with GeneDoc. A Neighbor-joining (NJ) phylogenetic tree was constructed using MEGA 6.0 software.

**Table 1:** Primers used in this study.

**Primer names**	**Sequence (5^**′**^-3^**′**^)**
IFITM1-AP1	GCAGTGGAGCCGTAGTGTC
IFITM1-AP2	GCGGGTAAACTGCTGGAT
IFITM1-SP1	CCACATCATCTGGTCCCTCT
IFITM1-SP2	CAGTTGCAGCATCCAGAATT
EcIFITM1-3HA-KpnI-F	CGGGGTACCATGAATCCAGCAGTTTACCC
EcIFITM1-3HA-XhoI-R	CCGCTCGAGTCAGTAACCATAATTGTACATGC
EcIFITM1-C1-KpnI-F	CGGGGTACCATGAATCCAGCAGTTTACCC
EcIFITM1-C1-BamHI-R	CGCGGATCCGTAACCATAATTGTACATGCTGT
EcRab5c-KpnI-F	CGGGGTACCATGGCAGGGCGAGGCGGA
EcRab5c-BamHI-R	CGCGGATCCGTTCCCGCCCCCACAGCA
EcRab7-KpnI-F	CGGGGTACCATGACTTCAAGGAAGAAAGTACTAC
EcRab7-BamHI-R	CGCGGATCCGCAGCTGCAGGTCTCTGC
siRNA1-EcIFITM1	GGAGGACAGTCAGTGGTTCAGTACA
siRNA2-EcIFITM1	CACCACTGTGAACGTCACCACTGAA
siRNA3-EcIFITM1	CCTCTTCATTGTACAGGCAGTTGCA
EcIFITM1-RT-F	CTGCTGCTGTGGCTTGTT
EcIFITM1-RT-R	ACACGGATGAGTTCCCTTT
β-actin-RT-F	TACGAGCTGCCTGACGGACA
β-actin-RT-R	GGCTGTGATCTCCTTCTGCA
SGIV MCP-RT-F	GCA CGCTTCTCTCACCTTCA
SGIV MCP-RT-R	AACGGCAACGGGAGCACTA
SGIV VP19-RT-F	TCCAAGGGAGAAACTGTAAG
SGIV VP19-RT-R	GGGGTAAGCGTGAAGAC
RGNNV CP-RT-F	CAACTGACAACGATCACACCTTC
RGNNV CP-RT-R	CAATCGAACACTCCAGCGACA
RGNNV RdRp-RT-F	GTGTCCGGAGAGGTTAAGGATG
RGNNV RdRp-RT-R	CTTGAATTGATCAACGGTGAACA
EcIRF7-RT-F	CAACACCGGATACAACCAAG
EcIRF7-RT-R	GTTCTCAACTGCTACATAGGG
EcISG15-RT-F	CCTATGACATCAAAGCTGACGAGAC
EcISG15-RT-R	GTGCTGTTGGCAGTGACGTTGTAGT
EcMXI-RT-F	CGAAAGTACCGTGGACGAGAA
EcMXI-RT-R	TGTTTGATCTGCTCCTTGACCAT
EcSPTssA-RT-F	CCCTCGGTGACTGTTGGA
EcSPTssA-RT-R	CCAGGGAGTTGAACACGGT
EcCers6-RT-F	TATTTTGGCGTGGTTTTGG
EcCers6-RT-R	CGTTGGGTTGTGCTTTCTG

### Expression Pattern of EcIFITM1 in Response to Viral Infection

To elucidate the changes in EcIFITM1 expression in response to fish virus infection *in vitro*, the relative expression of EcIFITM1 was detected by quantitative real-time PCR (qPCR) after SGIV or RGNNV infection, respectively. In brief, GS cells were infected with SGIV or RGNNV at a multiplicity of infection (MOI) of 2. The infected cells were harvested at 3, 6, 18, 24, and 30 h post-infection (h.p.i.) for qPCR analysis.

### Plasmid Construction

To elucidate the potential function of EcIFITM1 *in vitro*, EcIFITM1 was subcloned into pEGFP-C1 and pcDNA3.1-3 × HA using the primers listed in Table 1. Besides, Rab5 (early endosomes marker), or Rab7 (late endosomes marker) were subcloned into pDsRed2-C1 using the primers listed in Table 1. All of the constructed plasmids (pEGFP-EcIFITM1, HA-EcIFITM1, pDsRed2-Rab5, and pDsRed2-Rab7) were confirmed by DNA sequencing.

### Subcellular Localization

To determine the subcellular localization of EcIFITM1, GS cells were seeded into glass bottom cell culture dishes (35 mm), and co-transfected with 0.5 μg pEGFP-C1 or pEGFP-EcIFITM1 with 0.5 μg pDsRed2-ER (37), pDsRed2-Mito (42), pDsRed2-Rab5, or pDsRed2-Rab7. After 48 h post-transfection, the cells were fixed with 4% paraformaldehyde (PFA) for 1 h at room temperature, and stained with 4, 6-diamidino-2-phenylindole (DAPI) (Sigma-Aldrich, USA) for 5 min. Moreover, GS cells transfected with 1 μg pEGFP-C1 or pEGFP-EcIFITM1 for 48 h were stained with Lyso-Tracker (Red DND-99) (Invitrogen, USA; 1:1,000) for 5 min followed by staining with DAPI for 5 min. Finally, the cells were observed under confocal laser scanning microscope (CLSM; Carl Zeiss, Germany).

### Virus Infection Assay

To evaluate the effects of EcIFITM1 overexpression on viral replication, GS cells were transfected with pcDNA3.1-3 × HA or HA-EcIFITM1 for 24 h, followed by SGIV or RGNNV infection (MOI = 2), respectively. First, the cellular morphology was observed and photographed using a phase contrast microscope. Next, the virus-infected cells were harvested at 12 h.p.i. and 24 h.p.i. for qPCR and western blot. The SGIV-infected cells were collected at 24 h.p.i. and the viral titers were determined on GS cell monolayers by a 50% tissue culture infective dose (TCID_50_). In addition, the level of RGNNV-CP protein in the pcDNA3.1-3 × HA- or HA-EcIFITM1-overexpressing cells were evaluated by an indirect immunofluorescence assay as described below.

For the knockdown assay, three specific small interfering RNA (siRNA) oligonucleotides targeting different sequences of EcIFITM1 were designed and commercially synthesized by Invitrogen (Table 1). GS cells were transfected with the siRNAs (160 nM/well) or a negative control (NC) using Lipofectamine 2000 reagent (Invitrogen, USA) following the manufacturer's instructions as previously described (43). After 24 h post-transfection, the GS cells were infected with SGIV or RGNNV (MOI = 2), and collected at 24 h.p.i. for qPCR analysis.

### Virus Entry Assay

To determine the detailed roles of EcIFITM1 at the early stage of RGNNV or SGIV infection, qPCR assay and single-particle tracking technique were used to analyze the viral entry into the host cells. In one group, the GS cells were seeded into 24-well plates and transfected with HA-EcIFITM1 or EcIFITM1-specific siRNA for 24 h. The transfected cells were then infected with SGIV or RGNNV at 4°C to allow for synchronous virus adsorption into the cells. After 1 h, the GS cells were washed three times with cold serum-free medium to remove any unbound virus, after which a pre-warmed medium was added to the culture for another 4 h (SGIV) or 1 h (RGNNV) at 28°C. Non-internalized viruses were removed by washing the cells with citrate buffer (citric acid 40 mM, potassium chloride 10 mM, sodium chloride 135 mM, pH 3.0) for 1 min (12, 38). The cells were harvested to determine the amount of virus that had entered the cells by qPCR analysis.

In another experimental group, GS cells were grown in glass bottom cell culture dishes (35 mm) and transfected with pEGFP-EcIFITM1 or pEGFP-C1 for 24 h. The cells were pre-chilled at 4°C for 5 min and infected with purified SGIV particles labeled with Alex-Fluor 647 labeled SGIV (MOI = 10) as previously described (38, 39, 44, 45). The cells were observed under CLSM and photographed. In each sample, 30 cells were randomly selected for analysis. The data were represented as the mean ± standard error of the mean (SEM). Since it is difficult to obtain highly purified RGNNV particles (~25 nm in diameter) for confocal microscopy, the effects of EcIFITM1 on RGNNV entry were only measured using qPCR as described above (38).

### RNA Isolation and qPCR Analysis

Total RNA was extracted using an SV Total RNA Isolation Kit (Promega, USA) and reversed with a ReverTra Ace qPCR RT Kit (TOYOBO, Japan) according to the manufacturer's protocol as described previously (43). qPCR was performed in an Applied Biosystems QuantStudio 5 Real Time Detection System (Thermofisher, USA) with the primers listed in Table 1. Each assay was carried out in triplicate under the following cycling conditions: 95°C for 1 min for activation, followed by 40 cycles at 95°C for 15 s, 60°C for 15 s, and 72°C for 45 s. The expression level of viral, host IFN, and ceramide synthesis-related genes was normalized to β-actin and calculated using the 2^−ΔΔCT^ method. The data were represented as the mean ± standard deviation (SD).

### Western Blot Assay

The total cell proteins were extracted with Pierce RIPA Lysis buffer (Thermofisher, USA), and the protein concentration was measured using a BCA protein assay kit (Kaiji, China) according to the manufacturer's instructions. The extracted proteins were electrophoresed by 10% SDS-polyacrylamide gel electrophoresis (SDS-PAGE), transferred to polyvinylidene difluoride (PVDF) membranes (Millipore, USA) for 60 min, and blocked with 5% (w/v) skim milk in Tris-buffered saline (pH 7.4, TBS) containing 0.5% Tween 20. The separated proteins were incubated with the following specific primary antibodies: anti-HA (Sigma, USA; 1:1,000 dilution), anti-SGIV major capsid protein (MCP) (1:1,500 dilution) (prepared in our lab), anti-RGNNV capsid protein (CP) (1:1,500 dilution) (prepared in our lab), and anti-β-actin (Abcam, USA; 1:1,000 dilution) for 2 h at room temperature. The membrane was then incubated with horseradish peroxidase (HRP)-conjugated sheep-rabbit IgG or sheep-mouse IgG at a dilution of 1:5,000 (Abcam, USA) for 2 h at room temperature. Immunoblots were visualized using an enhanced HRP-DAB Substrate Chromogenic Kit (Tiangen, China) according to the manufacturer's protocol. The intensity of each protein was quantified using Image J software and normalized to the expression of β-actin.

### Indirect Immunofluorescence Assay (IFA)

GS cells transfected with pcDNA3.1-3 × HA or HA-EcIFITM1 were infected with RGNNV (MOI = 2) for IFA as previously described (46). Briefly, the RGNNV-infected GS cells were incubated with primary antibodies in anti-CP serum (1:200) diluted in 0.2% bovine serum albumin (BSA) for 2 h at room temperature. After washing three times with phosphate buffered saline (PBS), the GS cells were incubated with the secondary antibody anti-rabbit IgG Fab2 Alexa Fluor 488 (Invitrogen, USA; 1:200) at room temperature for 2 h. Finally, the cells were stained with 1 mg/mL DAPI and observed under an inverted fluorescence microscope.

### Non-targeted Lipometabolomics Analysis

Sample preparation: GS cells were grown in six-well cell culture plates at 28°C for 18 h, and transfected with pcDNA3.1-3 × HA or HA-EcIFITM1 for 36 h. The transfected GS cells (1 × 10^6^ cells/well; *n* = 6) were collected, and were centrifuged at 4°C, 300 × g for 10 min. After washing once with cold PBS, the cells were centrifuged at 4°C, 300 × g for 10 min. One well of the samples was collected for western blot analysis as described above and the rest were immediately frozen on ice and stored at −80°C until further use.

Metabolite extraction: After confirming that HA-EcIFITM1 was successfully overexpressed, the remaining cell pellets were subjected to metabolic extraction. A volume of 800 μL PBS was added to all of the samples, followed by an ultrasound at 40 kHz, 360 w for 5 min. After adding 600 μL methanol to precipitate the proteins, the samples were extracted with 400 μL methanol and 2 mL dichloromethane containing 10 μL internal standards (100 μg/mL), vortexed for 30 min, and centrifuged to separate the aqueous phase from the organic phase for further metabolomics analyses.

Ultra-performance liquid chromatography (UPLC)-QTOF-Mass spectrometry (MS) profiling analysis: A UPLC system used was Shimadzu UPLC lc-30a with Phenomenex Kinete C18 column (100 × 2.1 mm, 2.6 μm). Injection volume, 1 μL; flow rate, 0.4 mL/min; column temperature, 60°C; sample chamber temperature, 4°C. Phase A consisted of H_2_O, MeOH, and ACN at a ratio of 1:1:1 with 5 mM NH4Ac. Phase B consisted of IPA and CAN at a ratio of 1:1 with 5 mM NH4Ac. Gradient conditions were as follows: 0.5 min, 20% phase B; 1.5 min, 40% phase B; 3 min, 60% phase B; 13 min, 98% phase B; 13.1 min, 20% phase B; 17 min, 20% phase B. An MS system was AB Sciex TripleTOF® 6600, using electrospray ionization (ESI) under the positive ion modes. The mass scanning range was 100–1,200 m/z and the mass parameters were as follows: curtain gas, 35.00 psi; ion source gas 1, 50.00; ion source gas 2, 50.00; ionspray voltage, 5,500.00 V; and temperature, 600°C.

Data preprocessing and statistical analysis: The data was edited into a two-dimensional data matrix using Excel 2010 software, including the retention time (RT), mass to load ratio (M/Z), molecular formula of substance, and compounds. Principal components analysis (PCA) and orthogon partial least squares-discriminant analysis (OPLS-DA) were used to test the metabolic differences between the control and treatment groups. Differential metabolites were screened based on the variable importance in projection (VIP) (≥ 1) from an OPLS-DA model and absolute Log2-fold change (FC) (≥1) obtained from a two-tailed Student's *t*-test.

Metabolite extraction, UPLC-QTOF-MS, data preprocessing, and statistical analysis were completed using Wuhan MetWare Biotechnology Co., Ltd.

### Statistical Analysis

The statistical analyses were performed using SPSS version 20. A one-way ANOVA was used to evaluate the differences between groups. Differences were considered statistically significant when the *p*-value was < 0.05 (^*^p < 0.05).

## Results

### Characterization of EcIFITM1

**Figure 1:**
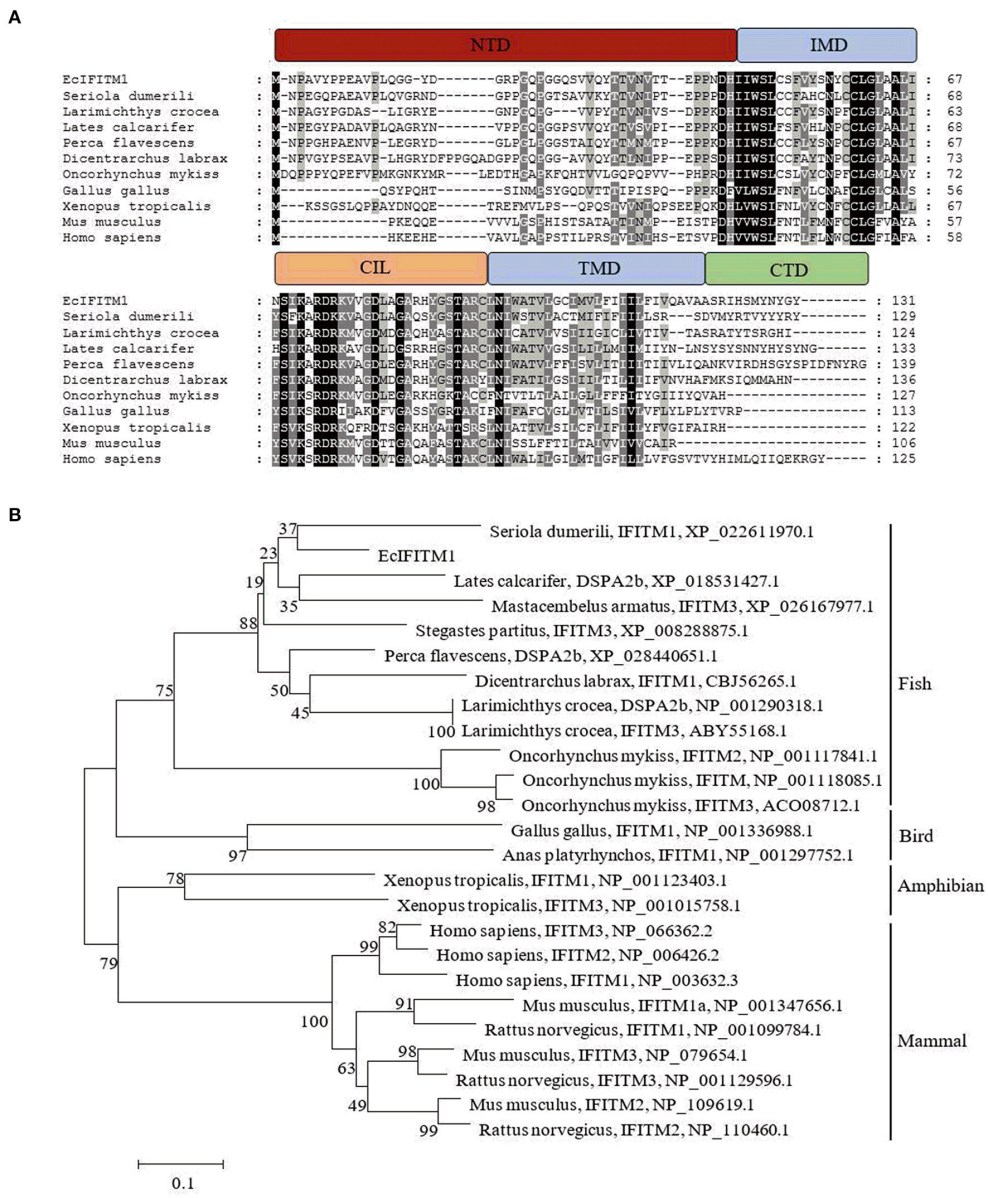
Analysis of IFITM1 proteins. **(A)** Multiple alignments of IFITM1 from different species. The NTD, IMD, CIL, TMD, and CTD domains were represented by the red, blue, orange, blue, and green bars, respectively. The accession numbers of IFITM1s were listed as follows: *E. coioides*, MW118611; *Seriola dumerili*, XP_022611970.1; *Larimichthys crocea*, NP_001290318.1; *Lates calcarifer*, XP_018531427.1; *Perca flavescens*, XP_028440651.1; *Dicentrarchus labrax*, CBJ56265.1; *Oncorhynchus mykiss*, NP_001118085.1; *Gallus gallus*, NP_001336988.1; *Xenopus tropicalis*, NP_001123403.1; *Mus musculus*, NP_001347656.1; *Homo sapiens*, NP_003632.3. **(B)** A neighbor-joining tree of IFITM1s. The GenBank accession number of the species was listed at the right of the species name and the numbers at the nodes denoted the bootstrap values of 1,000 replicates. The scale represents the number of substitutions per 1,000 bases.

Using RACE technique, we obtained the full-length cDNA sequence of EcIFITM1 based on the EST sequences from the grouper spleen transcriptome. EcIFITM1 contained a predicted ORF of 396 bp encoding 131 amino acids and flanked by 147 bp of the 5'-untranslated region (UTR) and 346 bp of the 3′-UTR with a poly (A) tail (GenBank Accession No: MW118611). The homology analysis results indicated that EcIFITM1 shared 64 and 43% identity with the IFITM1 homolog from greater amberjack, *S. dumerili* (XP_022611970.1), and humans, *Homo sapiens* (NP_003632.3), respectively. The amino acid alignment analysis indicated that EcIFITM1 contained five domains: (1) a variable hydrophobic N-terminal domain (NTD) (aa 1–45); (2) a conserved hydrophobic intramembrane domain (IMD) (aa 46–67); (3) a conserved intracellular loop (CIL) (aa 68–93); (4) a variable hydrophobic transmembrane domain (TMD) (aa 94–119); and (5) a highly variable C-terminal domain (CTD) (aa 120–131) (Figure 1A). The phylogenetic analysis showed that EcIFITM1 had the nearest phylogenetic relationship to *S. dumerili* in fish group, apart from bird, amphibian, and mammalian IFITMs (Figure 1B).

### Expression Pattern of EcIFITM1 *in vitro*

**Figure 2:**
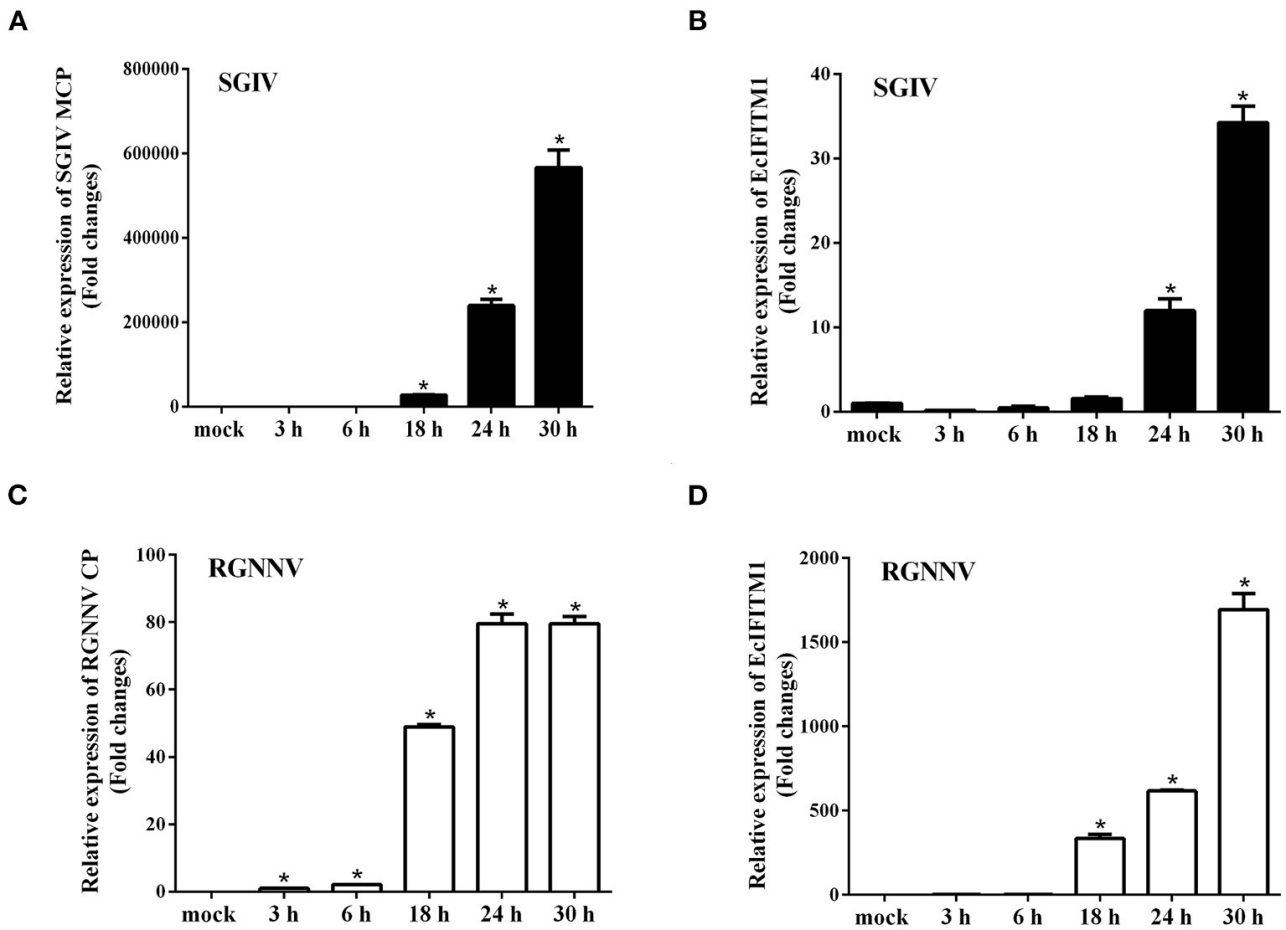
The expression profiles of EcIFITM1. **(A)** The level of SGIV MCP mRNA expression during SGIV infection. **(B,D)** The level of EcIFITM1 transcription in response to SGIV **(B)** or RGNNV **(D)** infection. **(C)** RGNNV CP expression during RGNNV infection. GS cells were infected with SGIV **(A,B)** or RGNNV **(C,D)** (MOI = 2) for 30 h, respectively, and were collected at the indicated time points for qPCR (*n* = 3; means ± SD). *p < 0.05.

To analyze the expression pattern of EcIFITM1 in response to different viruses, GS cells were infected with RGNNV or SGIV, and collected at the indicated time points for qPCR. As shown in Figure 2A, SGIV MCP was significantly induced by SGIV infection. Consistently, the level of EcIFITM1 transcription increased from 24 h.p.i. and peaked at 34-fold at 30 h.p.i. compared with the mock-infected cells (Figure 2B). Moreover, the level of RGNNV CP mRNA expression increased gradually during RGNNV infection (Figure 2C). EcIFITM1 was significantly induced by RGNNV and reached 1,694-fold at 30 h.p.i. (Figure 2D). Our results indicated that both fish DNA and RNA viruses could upregulate the expression of EcIFITM1.

### Cellular Localization of EcIFITM1

**Figure 3:**
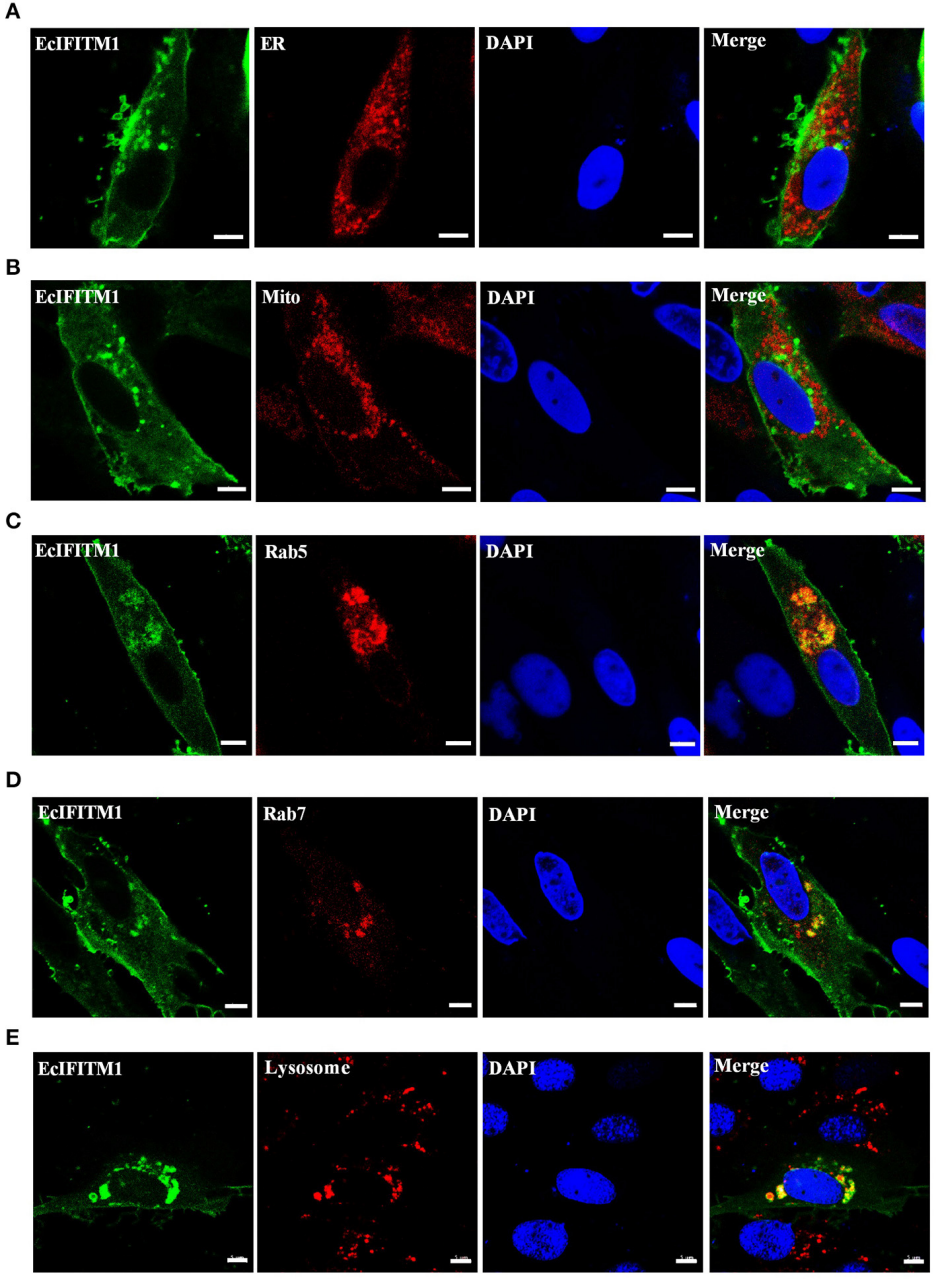
Cellular localization of EcIFITM1. GS cells were co-transfected with pEGFP-C1 or pEGFP-EcIFITM1 and pDsRed2-ER **(A)**, pDsRed2-Mito **(B)**, pDsRed2-Rab5 **(C)**, or pDsRed2-Rab7 **(D)** for 48 h. Moreover, GS cells transfected with pEGFP-C1 or pEGFP-EcIFITM1 for 48 h were dyed with Lyso-Tracker **(E)** for 5 min. The cells were imaged under fluorescence microscopy. Scale bars were 5 μm.

Several literatures demonstrate that IFITMs are localized in the early and late endosomes (47), lysosomes (LYs) (48), endoplasmic reticulum (ER), Golgi, and mitochondrion (Mito) (12) to restrict viral infection. To explore the cellular localization of EcIFITM1 *in vitro*, pEGFP-C1 or pEGFP-EcIFITM1 were co-transfected with pDsRed2-ER, pDsRed2-Mito, pDsRed2-Rab5, or pDsRed2-Rab7 into GS cells. Moreover, at 48 h post-transfection, GS cells transfected with pEGFP-C1 or pEGFP-EcIFITM1 were stained with Lyso-Tracker. As shown in Figure 3, the green fluorescence in pEGFP-EcIFITM1 transfected cells was primarily distributed in the cytoplasm, and accumulated in dots around the nucleus. Notably, the green fluorescence of EcIFITM1 was not co-localized with the red fluorescence of the ER (Figure 3A) and Mito (Figure 3B), but partly with that of Rab5 (Figure 3C), Rab7 (Figure 3D), or lysosomes (Figure 3E). Thus, our results suggested that EcIFITM1 encoded an endosomal- and lysosomal-localized protein.

### EcIFITM1 Inhibited SGIV and RGNNV Replication *in vitro*

**Figure 4:**
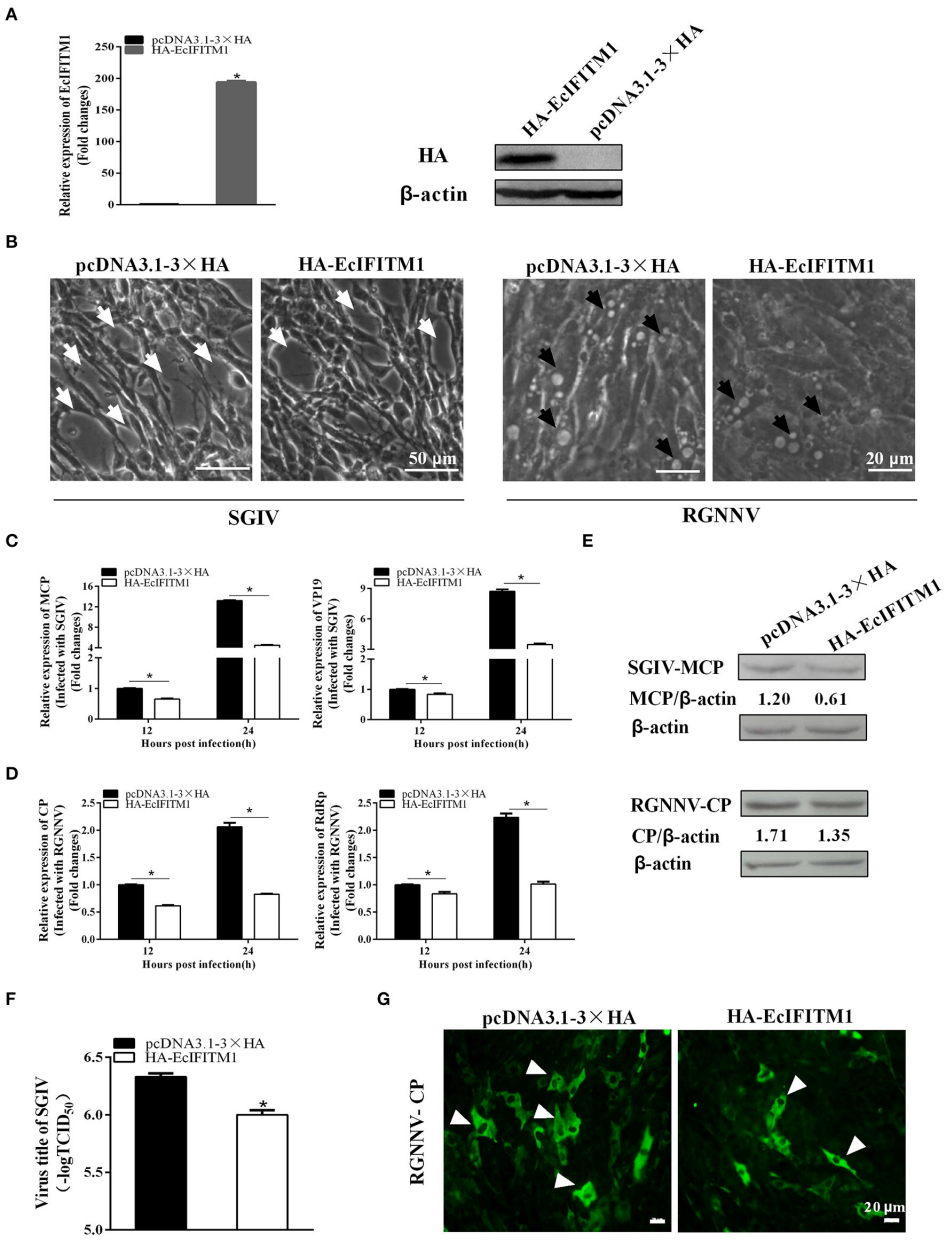
Roles of EcIFITM1 overexpression on SGIV and RGNNV replication. **(A)** The level of EcIFITM1 mRNA and protein expression in pcDNA3.1-3 × HA- or HA-EcIFITM1-overexpressing cells by qPCR and western blot. **(B)** EcIFITM1 overexpression weakened the CPE progression induced by SGIV and RGNNV. The white arrows showed the cell rounding and aggregation of cells evoked by SGIV infection. The black arrows indicated that the vacuoles were induced by RGNNV infection. **(C,D)** The level of viral gene transcription in GS cells transfected with pcDNA3.1-3 × HA or HA-EcIFITM1. Transfected GS cells were infected with SGIV or RGNNV, and harvested at 12 h.p.i. and 24 h.p.i. to determine the mRNA expression level of MCP and VP19 of SGIV **(C)** or CP and RdRp of RGNNV **(D)** by qPCR. **(E)** The level of SGIV-MCP and RGNNV-CP protein in transfected GS cells at 24 h.p.i. by western blot. **(F)** SGIV production in transfected GS cells. A TCID_50_ assay was used to measure the virus titer of SGIV. **(G)** The positive fluorescence signal of RGNNV-CP in transfected GS cells by IFA. Arrowheads showed the fluorescence signal of CP (*n* = 3; means ± SD). *p < 0.05.

To determine the roles of EcIFITM1 during fish virus infection, we evaluated the effects of EcIFITM1 overexpression on the replication of SGIV and RGNNV. After infection with SGIV or RGNNV in pcDNA3.1-3 × HA- or HA-EcIFITM1-transfected cells, virus replication including virus production, viral gene transcription and protein synthesis were examined by TCID_50_, qPCR, western blot, and IFA, respectively. First, the level of mRNA and protein expression of EcIFITM1 in HA-EcIFITM1- or pcDNA3.1-3 × HA-overexpressing cells was evaluated. The results showed that HA-EcIFITM1 was successfully overexpressed in transfected cells (Figure 4A). The severity of the cytopathic effects (CPE) induced by SGIV and RGNNV infection at 24 h.p.i. was obviously weakened in the EcIFITM1-overexpressing cells compared to that of the control vector cells (Figure 4B). As shown in Figures 4C,D, EcIFITM1 overexpression significantly inhibited the transcription level of SGIV MCP and VP19 (Figure 4C), as well as the RGNNV RNA-dependent RNA-polymerase (RdRp) and CP genes (Figure 4D). Consistently, the level of SGIV-MCP and RGNNV-CP protein expression were also decreased in the EcIFITM1-transfected cells compared to that of the control cells at 24 h.p.i. (Figure 4E). The virus titer assay indicated that the production of SGIV was significantly reduced in HA-EcIFITM1-transfected cells compared with the control cells (Figure 4F). In addition, the IFA results demonstrated that EcIFITM1 overexpression significantly decreased RGNNV infected cells, indicated by the reduced positive fluorescence signal of the RGNNV-CP protein (Figure 4G).

**Figure 5:**
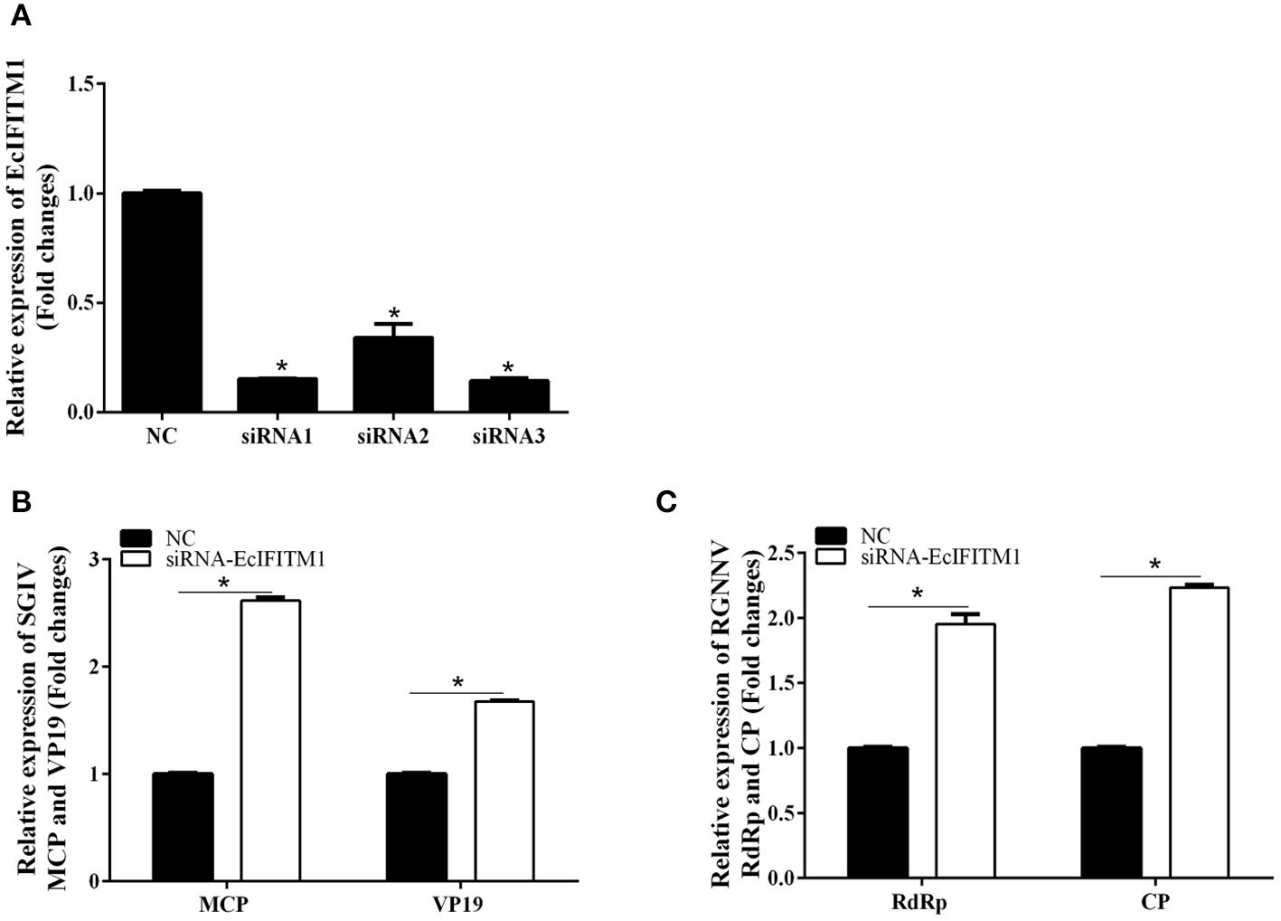
The effects of EcIFITM1 knockdown on SGIV and RGNNV replication. **(A)** The interference efficiency of three specific siRNAs by qPCR. **(B,C)** A knockdown of EcIFITM1 increased the level of SGIV and RGNNV gene transcription. GS cells transfected with NC or specific siRNA-EcIFITM1 were infected with SGIV or RGNNV, and collected at 24 h.p.i. for qPCR analysis of the expression of SGIV MCP and VP19 **(B)**, as well as RGNNV CP and RdRp **(C)** (*n* = 3; means ± SD). *p < 0.05.

We subsequently investigated the effect of EcIFITM1 knockdown on the replication of SGIV and RGNNV. As shown in Figure 5A, compared with the NC siRNA, three specific siRNAs targeting EcIFITM1 all significantly inhibited EcIFITM1 expression with 85, 66, and 86% knockdown efficiency, respectively. Thus, siRNA3-EcIFITM1 was chosen for subsequent experiments. As shown in Figure 5B, EcIFITM1 knockdown by siRNA3 increased the levels of SGIV MCP and VP19 transcription. Moreover, the transcription of the RGNNV RdRp and CP genes was significantly increased in siRNA-EcIFITM1 transfected cells compared with that of NC siRNA transfected cells (Figure 5C). Taken together, it was proposed that EcIFITM1 served as an antiviral effector in response to fish DNA and RNA viruses infection.

### EcIFITM1 Restricted SGIV and RGNNV Entry

**Figure 6:**
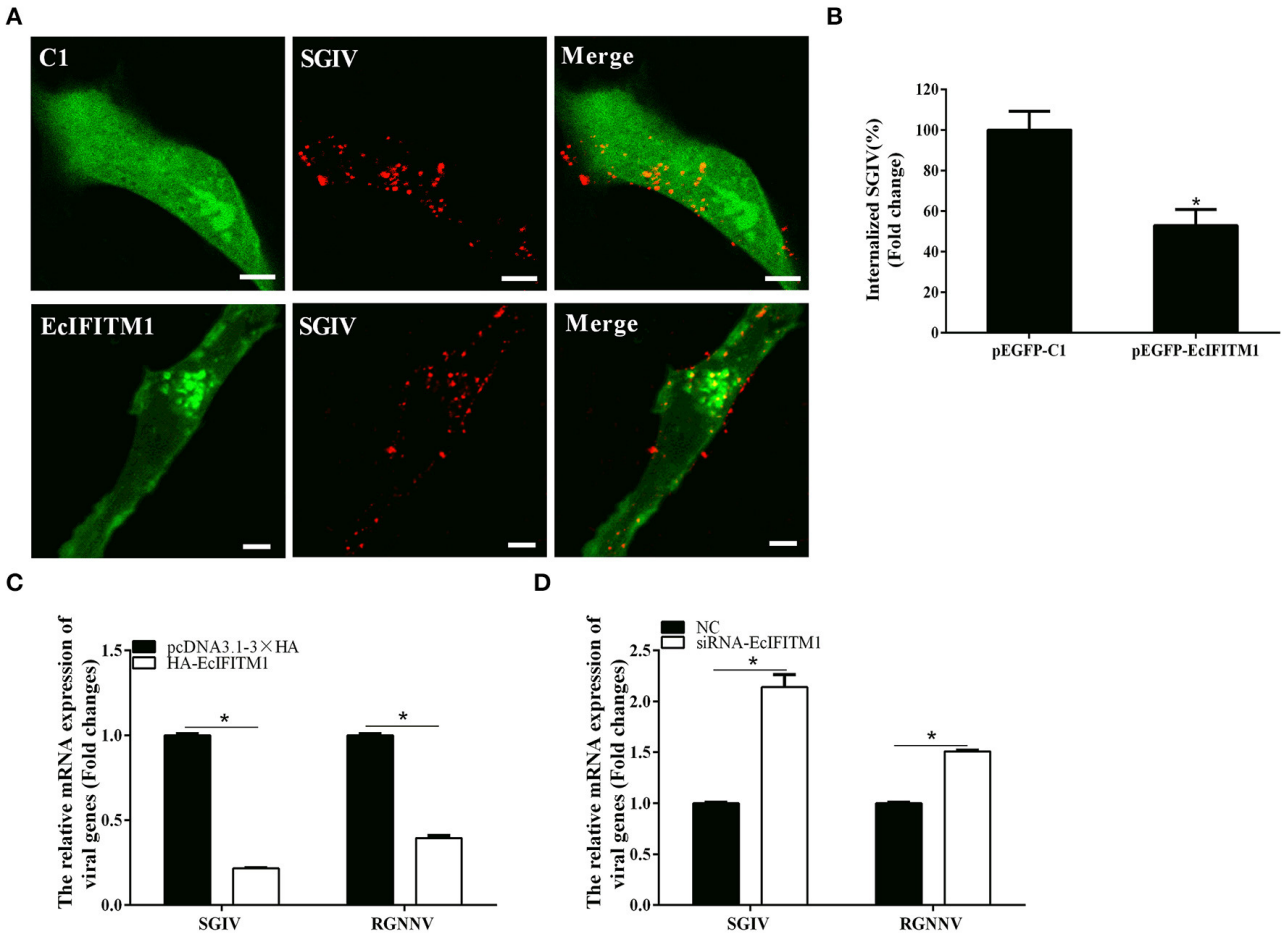
EcIFITM1 inhibited SGIV and RGNNV entry. **(A)** The overexpression of EcIFITM1 reduced the number of SGIV in GS cells. **(B)** The quantification of the internalized SGIV particles in pEGFP-C1- or pEGFP-EcIFITM1-overexpressing cells. Transfected GS cells (green) were infected with Alex-Fluor 647 labeled SGIV (red) for 1 h and imaged by CLSM. Internalized SGIV in pEGFP-EcIFITM1-overexpressing cells was normalized to that in the empty vector cells. Scale bars were 5 μm. Data were expressed as the mean ± SEM from three independent experiments (*n* = 30). **(C)** EcIFITM1 overexpression decreased the mRNA expression of SGIV MCP and RGNNV CP in GS cells. **(D)** A knockdown of EcIFITM1 increased the level of SGIV MCP and RGNNV CP transcription in GS cells. GS cells transfected with HA-EcIFITM1 or specific siRNA-EcIFITM1 were infected with SGIV (4 h) or RGNNV (1 h), and collected at the indicated time points to detect the relative expression of viral genes in the cytoplasm by qPCR (*n* = 3; means ± SD). *p < 0.05.

To explore whether EcIFITM1 acted at the early stages of SGIV and RGNNV infection, we performed virus entry assay using a confocal microscope and qPCR analysis (12, 38). Using Alex-Fluor 647-labeled SGIV, we observed the number of internalized SGIV particles in the cytoplasm and quantified using CLSM. As shown in Figures 6A,B, EcIFITM1 overexpression significantly reduced the number of red-fluorescence-labeled SGIV in the cytoplasm (52.8% of control value) compared with the control vector transfected cells. Consistently, the level of SGIV MCP and RGNNV CP transcription in the cytoplasm were significantly decreased in EcIFITM1-overexpressing cells (Figure 6C). In addition, compared with the negative control cells, knockdown of EcIFITM1 significantly increased the level of mRNA expression of viral genes in the cytoplasm (Figure 6D). Together, these results indicated that EcIFITM1 might affect virus replication by inhibiting the entry of SGIV and RGNNV *in vitro*.

### EcIFITM1 Overexpression Regulated Lipid Metabolism

**Figure 7:**
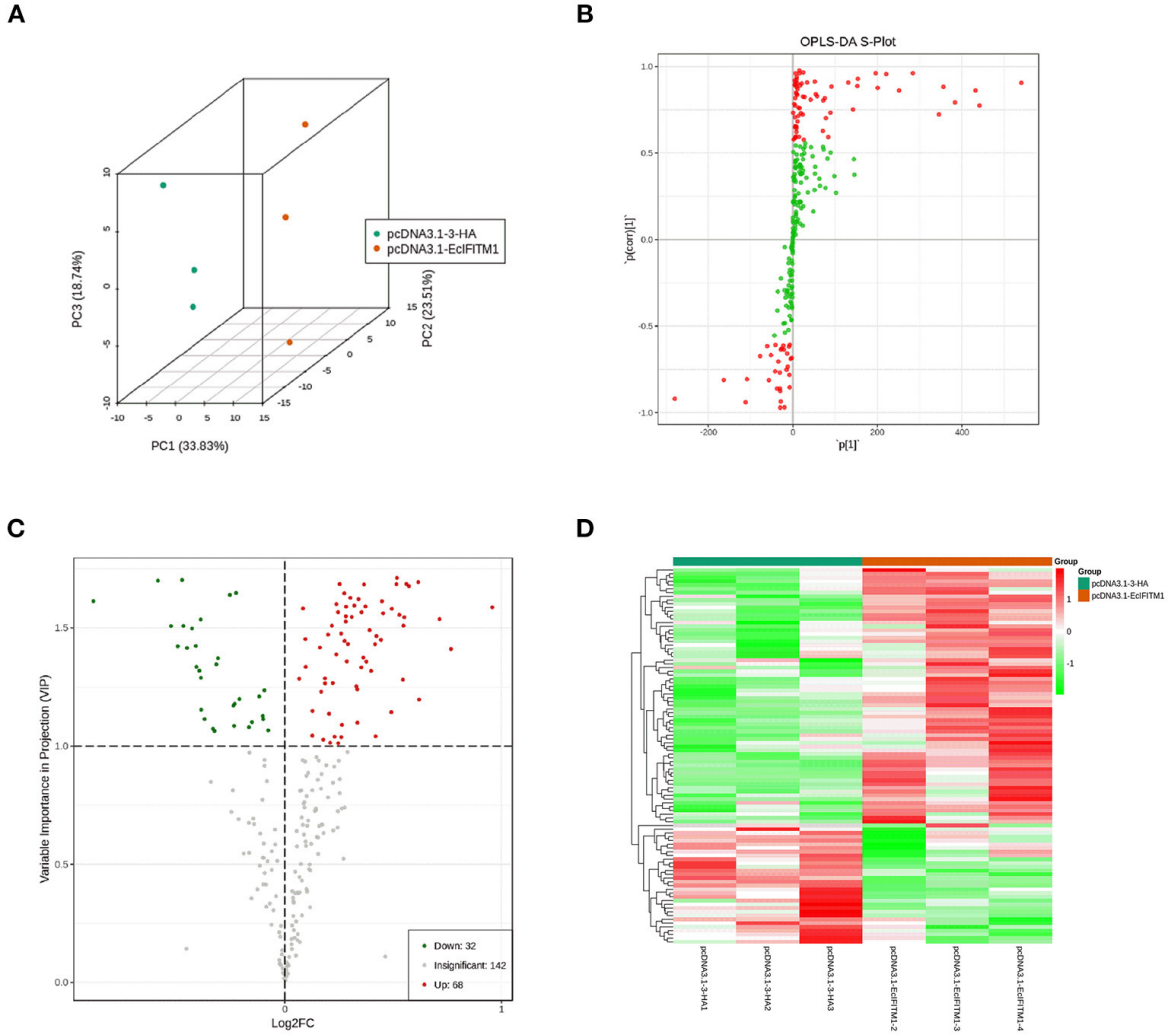
EcIFITM1 overexpression regulated lipid metabolism. **(A)** An analysis of PCA score plots (three-dimensional). **(B)** S-plot of OPLS-DA. **(C)** Volcano map. The red dots indicated that the VIP value of these metabolites was ≥1. The green dots indicated that the VIP value of these metabolites was <1. The gray dots indicated that the metabolite was not significantly changed. **(D)** Cluster heat map of the differential metabolites.

The cell lipid membrane forms a barrier, which closely regulates the entry and egress of many viruses. It has been reported that IFITMs blocked viral entry by altering intracellular cholesterol homeostasis (48). To determine whether EcIFITM1 could regulate the changes in the cell lipid composition that affected fish virus entry, GS cells transfected with HA-EcIFITM1 or pcDNA3.1-3 × HA were collected to measure the changes in lipid metabolites by UPLC-QTOF-MS. As shown in Supplementary Table 1, 242 metabolites were detected based on the UPLC-QTOF-MS platform and database. First, the clustering and differences between the control groups and the EcIFITM1-overexpressing groups were revealed by multivariate statistical approaches, including PCA and OPLS-DA. The PCA results showed that there were significant differences between the control groups and EcIFITM1-overexpressing groups (Figure 7A). In addition, the quality parameters of the OPLS-DA model, with R^2^X = 0.521, R^2^Y = 0.909, and *Q*^2^ = 0.798, indicated that the lipid metabolic profiles in the EcIFITM1-overexpressing cells were significantly altered compared to the control cells (Figure 7B). According to the two screening criteria (VIP ≥ 1 and absolute Log2 FC ≥ 1), compared with control cells, 100 differential metabolites were screened and identified in pcDNA3.1-EcIFITM1-overexpressing cells (Supplementary Table 2). Among these metabolites, the level of 68 metabolites were up-regulated and 32 metabolites were down-regulated (Figure 7C). Using hierarchical cluster analysis, we evaluated the variation characteristics of the metabolites between pcDNA3.1-EcIFITM1 and pcDNA3.1-HA transfected cells. The clustering results were shown in the heat map in Figure 7D, and displayed significantly different levels between pcDNA3.1-EcIFITM1 and pcDNA3.1-HA groups. Furthermore, the screened differential metabolites were primarily divided into the following three categories: sphingolipids, fatty acids, and glycerophospholipids without cholesterol (Table 2). It is important to note that 18 of the detected ceramides were significantly up-regulated, which suggested that ceramides play an important role in the antiviral function of EcIFITM1.

**Table 2:** Differential lipid metabolites in EcIFITM1-overexpressing cells.

**Full name**	**Abbreviation**	**Total numbers (detected)**	**Number of up-regulated**	**Number of down-regulated**	**Classification**
Ceramide	cer	18	18	0	Sphingolipid
Free fatty acids	FFA	13	7	6	Fat
Lysophosphatidylethanolamine	LPE	4	4	0	Glycerin phospholipid
Phosphatidylethanolamine	PE	16	9	7	Glycerin phospholipid
Phosphatidylglycerol	PG	5	3	2	Glycerin phospholipid
Phosphatidylinositol	PI	2	1	1	Glycerin phospholipid
Lysophosphatidylcholine	LPC	1	0	1	Glycerin phospholipid
Phosphatidylcholine	PC	31	25	6	Glycerin phospholipid
Sphingomyelin	SM	4	0	4	Sphingolipid
Triglyceride	TAG	6	1	5	Fat

**Figure 8:**
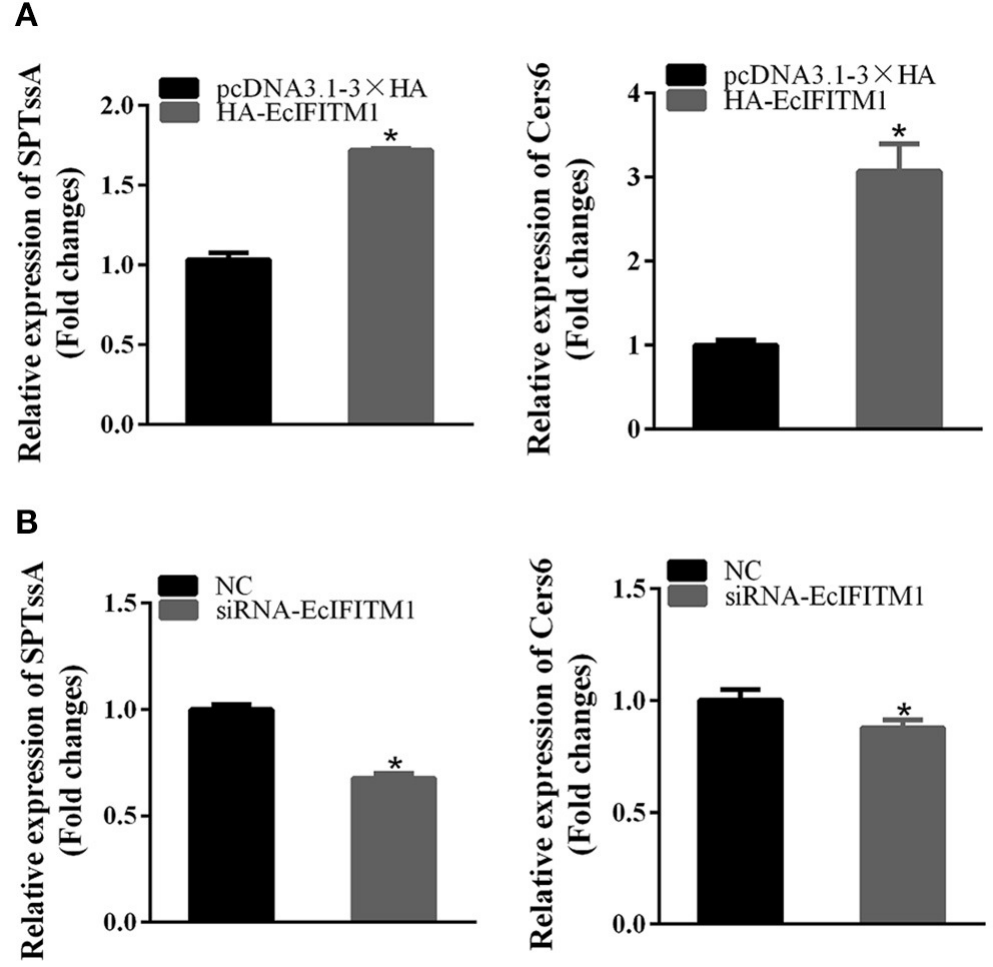
EcIFITM1 was involved in the regulation of ceramide synthesis. GS cells were transfected with HA-EcIFITM1 or siRNA-EcIFITM1 for 48 h, and were collected to detect the relative expression of ceramide synthesis-related genes, including SPTssA and Cers6 by qPCR in EcIFITM1-overexpressing **(A)**, or EcIFITM1-silenced cells **(B)** (*n* = 3; means ± SD). *p < 0.05.

A previous study showed that ceramide produced through *de novo* biosynthesis pathway which is catalyzed by serine palmitoyl transferase (SPT) and ceramide synthase (Cers) played an antiviral role in IAV infection (49). To further clarify the function of EcIFITM1 on ceramide synthesis, we tested the mRNA expression of two genes related to ceramide synthesis by qPCR, including SPTssA, and Cers6. As shown in Figure 8, the level of SPTssA and Cers6 transcription were increased by the ectopic expression of EcIFITM1 (Figure 8A), but decreased by EcIFITM1 silencing (Figure 8B). Together, these findings indicated that EcIFITM1 might be involved in the regulation of ceramide synthesis.

### EcIFITM1 Positively Regulated the IFN Immune Response

**Figure 9:**
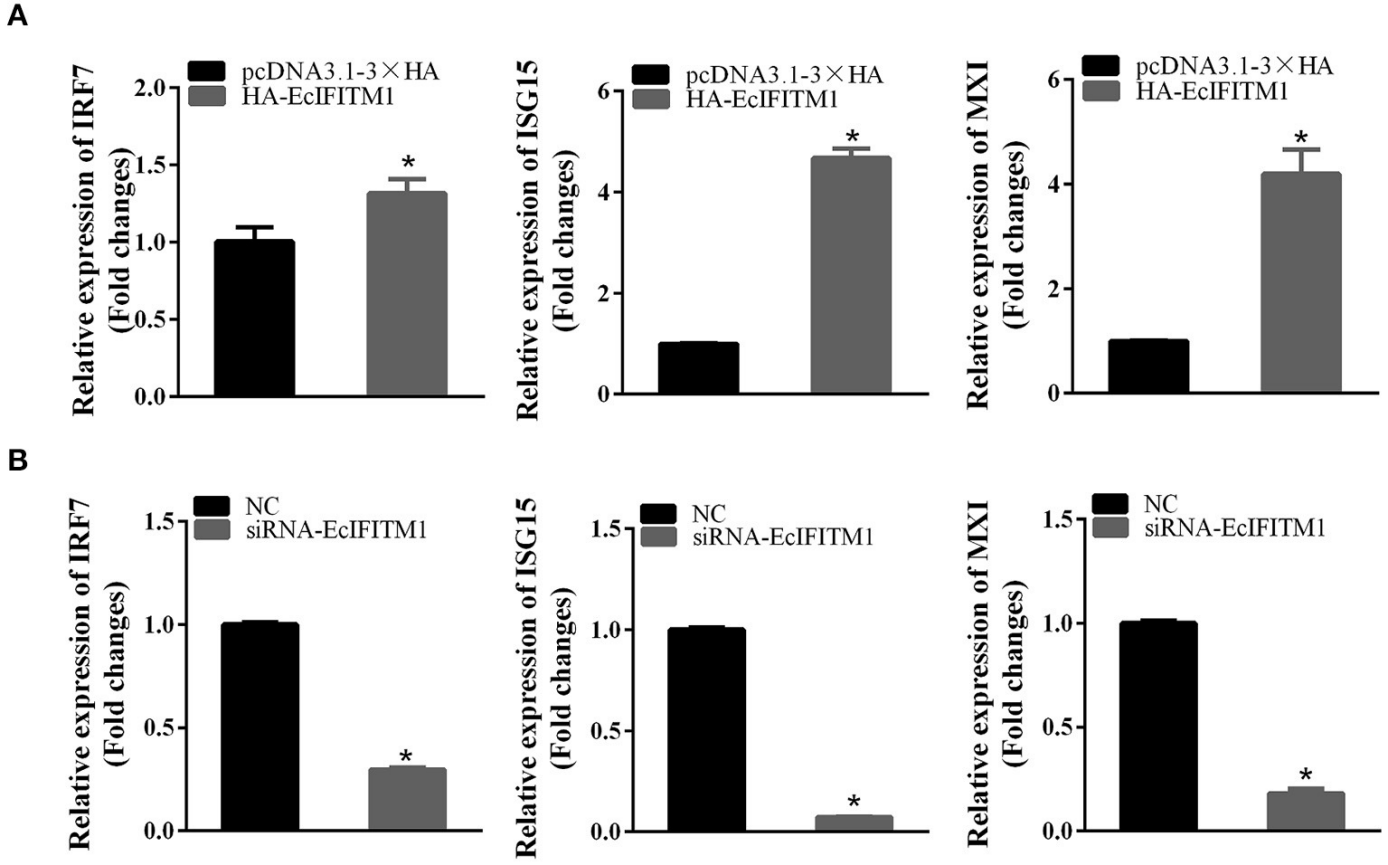
EcIFITM1 positively regulated the immune response. The level of interferon signaling molecule mRNA, including IRF7, ISG15, and MXI in EcIFITM1-overexpressing **(A)** or EcIFITM1-silenced cells **(B)** were determined by qPCR (*n* = 3; means ± SD). *p < 0.05.

To elucidate the effects of EcIFITM1 on the host IFN immune response, the level of mRNA expression of host IFN molecules was examined by qPCR. As demonstrated in Figure 9A, the mRNA expression of several IFN-related genes, including IRF7, ISG15, and myxovirus resistance gene (MX) I, were significantly increased in the EcIFITM1-overexpressing cells. Consistently, knockdown of EcIFITM1 significantly down-regulated the expression levels of these IFN-related genes (Figure 9B). Thus, it was speculated that EcIFITM1 positively regulated the host interferon immune response.

## Discussion

IFITM proteins, including IFITM1, IFITM2, and IFITM3, are highly induced by type I and II interferons, and most cells express a basal level of one or more of these proteins in the majority of vertebrates. IFITM proteins have been reported to broadly inhibit both RNA and DNA virus infection, especially during the virus entry into host cells (47). However, few studies have focused on the antiviral function of IFITM1 on fish viruses.

EcIFITM1 encoded a 131-amino-acid polypeptide which shared 64% identity with an IFITM1 homolog from *S. dumerili*. Bioinformatic analysis showed that EcIFITM1 contained all the 5 conserved domains, including NTD, IMD, CIL, TMD, and CTD. Among them, NTD, IMD and CIL have been demonstrated to be critical for regulating the subcellular localization and antiviral activity of IFITM proteins (50, 51). After infection of SGIV or RGNNV *in vitro*, the expression level of EcIFITM1 was significantly increased. This is consistent with previous reports that IFITM1 was significantly up-regulated by different viruses, including RGV, SMRV (12), PRV (17), IAV H5N1 (52), and Kaposi's sarcoma-associated herpesvirus (KSHV) (53). Thus, we proposed that EcIFITM1 might be involved in fish virus infection and exert antiviral roles like mammalian IFITMs.

Previous studies have extensively described the antiviral function of IFITM1 on RNA viruses; however, few have focused on its roles in DNA viruses (4, 13, 54). Of note, our results showed that EcIFITM1 overexpression not only restricted the mRNA and protein expression of RGNNV, but also that of SGIV, a DNA virus of the *Iridoviridae* family. Moreover, knockdown of EcIFITM1 significantly promoted RGNNV and SGIV replication. In accordance with this effect, the level of SMRV and RGV production and transcription were significantly reduced by the ectopic expression of IFITM1, but enhanced by a knockdown of IFITM1 (12). In addition, IFITM1 overexpression inhibited the replication of a wide range of RNA viruses, including RSV, mumps virus, parainfluenza virus (PIV), human metapneumovirus (HMPV), Newcastle disease virus (NDV), and a DNA virus, herpes simplex virus (HSV) 1 (13), suggesting that the antiviral function of IFITM1 is conserved from lower vertebrates to mammals. In addition, a growing number of studies have demonstrated that IFITM1 can broadly limit viral infection, particularly at the step of virus entry (9). For example, human IFITM1 inhibited HCV entry by interacting with CD81 and occludin, which are both HCV co-receptors (55). IFITMs can also potentially trap the virions in the endosomal pathway to limit HCV infection (47). Similarly, our results showed that EcIFITM1 overexpression blocked SGIV and RGNNV entry. Moreover, EcIFITM1 was partly colocalized with some membrane organelles, including EEs, LEs, and LYs, which was in agreement with previous studies in mammals (47). Our previous study demonstrated that SGIV particles were co-localized with EEs, LEs, and LYs and were transported along with these organelles during the early stage of infection (44). Thus, we hypothesized that EcIFITM1 might trap the virus in the endosomal compartments to interfere with SGIV and RGNNV infection. However, the detailed mechanism by which EcIFITM1 restricts SGIV and RGNNV infection by targeting endosomal compartments requires further investigation.

The cell lipid membrane forms a barrier, closely regulating the entry and egress of several viruses. In addition, IFITMs inhibited virus entry by altering intracellular cholesterol homeostasis (48). To elucidate the effects of EcIFITM1 on lipid metabolism, we used UPLC-QTOF-MS to determine the changes in intracellular lipid metabolites within EcIFITM1 overexpressing cells. We found that EcIFITM1 overexpression significantly regulated the level of 100 differential metabolites, including sphingolipids, fatty acids, and glycerophospholipids. Of note, all the 18 detected ceramides, which is a central intermediate in sphingolipid metabolism, were significantly up-regulated. In addition, the level of mRNA of two ceramide synthesis-related genes were increased in EcIFITM1 overexpressing cells. Eukaryotic cell plasma membrane lipids consist primarily of sphingolipids, glycerophospholipids, and cholesterol, which play essential roles in every stage of the viral life cycle (56, 57). For example, ceramide accumulated by the *de novo* biosynthesis pathway has antiviral function against IAV infection (49). Thus, it is speculated that EcIFITM1 overexpression increased lipid metabolites to inhibit virus infection. The detailed mechanism by which EcIFITM1 is involved in lipid metabolism needed further verification.

It has been reported that IFITMs are involved in various cellular processes, which has both direct and indirect effects on immunity (58). A recent study showed that the expression levels of IRF1, human leucocyte antigen (HLA)-B, and ISG15 protein were attenuated in the IFITM1/IFITM3 double-null cells (59). Consistently, the ectopic expression of EcIFITM1 significantly increased the levels of IRF7, ISG15, and MXI mRNA, while knockdown of EcIFITM1 displayed an opposite effect. Moreover, these interferon signaling-related genes have been demonstrated to display antiviral activity against SGIV or RGNNV infection (35, 43, 60). For example, IRF7 overexpression inhibited SGIV replication, and ISG15 was able to decrease RGNNV infection in grouper cells. Thus, we proposed that IFITM1 might positively regulate the immune response to restrict SGIV and RGNNV infection.

In conclusion, an IFITM1 homolog from orange spotted grouper (EcIFITM1) was cloned and its role in fish virus infection were investigated in this study. EcIFITM1 encoded a cytoplasmic protein, and partly colocalized with EEs, LEs, and LYs. Moreover, the ectopic expression or knockdown of EcIFITM1 *in vitro* consistently indicated that EcIFITM1 exerted antiviral roles against RGNNV and SGIV infection, especially at the stage of virus entry. The antiviral action was due to the bi-functional regulatory roles on host lipid metabolism and immune response during virus infection. Together, our results will provide new insights into understanding the functions of IFITM1 against virus infections.

## Data Availability Statement

The datasets generated in this study can be found in online repositories. The names of the repository/repositories and accession number(s) can be found in the article/Supplementary Material.

## Author Contributions

YZ completed the main experiments, analyzed the data, and wrote the manuscript. LW, JZ, LH, SW, and XH participated in the preparation of reagents and virus stocks, and confocal microscopy analysis. QQ and YH conceived and supervised the study, and edited and reviewed drafts of the manuscript. All authors contributed to the article and approved the submitted version.

## Conflict of Interest

The authors declare that the research was conducted in the absence of any commercial or financial relationships that could be construed as a potential conflict of interest.
