# Supplementary material for: Grouper Interferon-Induced Transmembrane Protein 1 Inhibits Iridovirus and Nodavirus Replication by Regulating Virus Entry and Host Lipid Metabolism
Source: Front Immunol. 2021 Mar 9;12:636806. doi: 10.3389/fimmu.2021.636806 (PMC7985356; doi:10.3389/fimmu.2021.636806)
Supplement: Supplementary file 2 [file Table_2.docx]

Supplemental table 2 Differential metabolites in EcIFITM1-overexpressing cells

| Index | Rt(min) | m/z | Formula | Compounds | VIP | Fold_Change | Type |
| --- | --- | --- | --- | --- | --- | --- | --- |
| L1 | 4.01E+00 | 5.54E+02 | C31H61NO3 | Cer-NS d31:1; Cer-NS d17:1/14:0 | 1.24E+00 | 1.26E+00 | up |
| L2 | 4.00E+00 | 5.69E+02 | C32H63NO3 | Cer-NS d32:1; Cer-NS d18:1/14:0 | 1.47E+00 | 1.34E+00 | up |
| L3 | 4.35E+00 | 5.83E+02 | C33H65NO3 | Cer-NS d33:1; Cer-NS d17:1/16:0 | 1.14E+00 | 1.15E+00 | up |
| L4 | 4.35E+00 | 5.97E+02 | C34H67NO3 | Cer-NS d34:1; Cer-NS d18:1/16:0 | 1.56E+00 | 1.37E+00 | up |
| L5 | 4.08E+00 | 5.95E+02 | C34H65NO3 | Cer-NS d34:2; Cer-NS d18:2/16:0 | 1.36E+00 | 1.30E+00 | up |
| L6 | 4.55E+00 | 6.11E+02 | C35H69NO3 | Cer-NS d35:1; Cer-NS d18:1/17:0 | 1.58E+00 | 1.06E+00 | up |
| L7 | 4.76E+00 | 6.25E+02 | C36H71NO3 | Cer-NS d36:1; Cer-NS d18:1/18:0 | 1.49E+00 | 1.31E+00 | up |
| L8 | 4.43E+00 | 6.23E+02 | C36H69NO3 | Cer-NS d36:2; Cer-NS d18:2/18:0 | 1.57E+00 | 1.19E+00 | up |
| L9 | 5.67E+00 | 6.81E+02 | C40H79NO3 | Cer-NS d40:1; Cer-NS d18:1/22:0 | 1.62E+00 | 1.27E+00 | up |
| L10 | 5.22E+00 | 6.79E+02 | C40H77NO3 | Cer-NS d40:2; Cer-NS d18:1/22:1 | 1.48E+00 | 1.20E+00 | up |
| L11 | 5.44E+00 | 6.93E+02 | C41H79NO3 | Cer-NS d41:2; Cer-NS d17:1/24:1 | 1.71E+00 | 1.43E+00 | up |
| L12 | 6.16E+00 | 7.09E+02 | C42H83NO3 | Cer-NS d42:1; Cer-NS d18:1/24:0 | 1.69E+00 | 1.53E+00 | up |
| L13 | 5.67E+00 | 7.07E+02 | C42H81NO3 | Cer-NS d42:2; Cer-NS d18:1/24:1 | 1.43E+00 | 1.33E+00 | up |
| L14 | 5.30E+00 | 7.05E+02 | C42H79NO3 | Cer-NS d42:3; Cer-NS d18:2/24:1 | 1.59E+00 | 1.29E+00 | up |
| L16 | 6.16E+00 | 7.21E+02 | C43H83NO3 | Cer-NS d43:2; Cer-NS d19:2/24:0 | 1.68E+00 | 1.29E+00 | up |
| L17 | 6.67E+00 | 7.37E+02 | C44H87NO3 | Cer-NS d44:1; Cer-NS d18:1/26:0 | 1.37E+00 | 1.29E+00 | up |
| L18 | 6.17E+00 | 7.35E+02 | C44H85NO3 | Cer-NS d44:2; Cer-NS d18:1/26:1 | 1.25E+00 | 1.26E+00 | up |
| L19 | 5.78E+00 | 7.33E+02 | C44H83NO3 | Cer-NS d44:3; Cer-NS d20:2/24:1 | 1.57E+00 | 1.28E+00 | up |
| L21 | 2.04E+00 | 2.55E+02 | HC16H31O2 | FFA 16:0 | 1.42E+00 | 7.31E-01 | down |
| L22 | 1.11E+00 | 2.51E+02 | HC16H27O2 | FFA 16:2 | 1.53E+00 | 1.22E+00 | up |
| L23 | 2.31E+00 | 2.69E+02 | HC17H33O2 | FFA 17:0 | 1.15E+00 | 7.65E-01 | down |
| L24 | 2.60E+00 | 2.83E+02 | HC18H35O2 | FFA 18:0 | 1.18E+00 | 8.52E-01 | down |
| L26 | 1.83E+00 | 2.79E+02 | HC18H31O2 | FFA 18:2 | 1.27E+00 | 1.14E+00 | up |
| L27 | 3.10E+00 | 3.11E+02 | HC20H39O2 | FFA 20:0 | 1.29E+00 | 7.65E-01 | down |
| L28 | 2.70E+00 | 3.09E+02 | HC20H37O2 | FFA 20:1 | 1.58E+00 | 1.41E+00 | up |
| L29 | 3.59E+00 | 3.39E+02 | HC22H43O2 | FFA 22:0 | 1.37E+00 | 8.07E-01 | down |
| L31 | 2.83E+00 | 3.35E+02 | HC22H39O2 | FFA 22:2 | 1.59E+00 | 1.25E+00 | up |
| L32 | 2.52E+00 | 3.33E+02 | HC22H37O2 | FFA 22:3 | 1.61E+00 | 1.36E+00 | up |
| L37 | 4.21E+00 | 3.81E+02 | HC25H49O2 | FFA 25:0 | 1.20E+00 | 8.65E-01 | down |
| L39 | 4.03E+00 | 3.93E+02 | HC26H49O2 | FFA 26:1 | 1.65E+00 | 1.21E+00 | up |
| L40 | 3.73E+00 | 3.91E+02 | HC26H47O2 | FFA 26:2 | 1.28E+00 | 1.05E+00 | up |
| L41 | 2.27E+00 | 4.80E+02 | C2O4H7NPC21H41O3 | LPE 18:0 | 1.69E+00 | 1.43E+00 | up |
| L42 | 2.46E+00 | 4.64E+02 | C2O4H7NPC21H41O2 | LPE 18:1e | 1.45E+00 | 1.36E+00 | up |
| L43 | 2.05E+00 | 4.62E+02 | C2O4H7NPC21H39O2 | LPE 18:2e | 1.33E+00 | 1.28E+00 | up |
| L44 | 2.43E+00 | 5.08E+02 | C2O4H7NPC23H45O3 | LPE 20:0 | 1.04E+00 | 1.20E+00 | up |
| L46 | 4.04E+00 | 6.62E+02 | C2O4H7NPC33H63O4 | PE 30:0; PE 14:0-16:0 | 1.09E+00 | 1.20E+00 | up |
| L51 | 4.87E+00 | 6.77E+02 | C2O4H7NPC35H69O3 | PE 32:0e; PE 16:0e/16:0 | 1.50E+00 | 7.43E-01 | down |
| L55 | 4.29E+00 | 7.03E+02 | C2O4H7NPC36H67O4 | PE 33:1; PE 16:0-17:1 | 1.21E+00 | 9.21E-01 | down |
| L59 | 4.48E+00 | 7.17E+02 | C2O4H7NPC37H69O4 | PE 34:1; PE 16:0-18:1 | 1.24E+00 | 9.37E-01 | down |
| L73 | 4.79E+00 | 7.27E+02 | C2O4H7NPC39H71O3 | PE 36:3e; PE 18:2e/18:1 | 1.11E+00 | 9.33E-01 | down |
| L74 | 4.03E+00 | 7.39E+02 | C2O4H7NPC39H67O4 | PE 36:4; PE 18:1-18:3 | 1.01E+00 | 1.19E+00 | up |
| L77 | 4.81E+00 | 7.57E+02 | C2O4H7NPC40H73O4 | PE 37:2; PE 18:1-19:1 | 1.09E+00 | 8.50E-01 | down |
| L78 | 5.31E+00 | 7.43E+02 | C2O4H7NPC40H75O3 | PE 37:2e; PE 19:1e/18:1 | 1.04E+00 | 1.09E+00 | up |
| L80 | 4.02E+00 | 7.51E+02 | C2O4H7NPC40H67O4 | PE 37:5; PE 17:1-20:4 | 1.14E+00 | 1.41E+00 | up |
| L82 | 4.21E+00 | 7.35E+02 | C2O4H7NPC40H67O3 | PE 37:6e; PE 17:2e/20:4 | 1.15E+00 | 1.09E+00 | up |
| L83 | 4.03E+00 | 7.33E+02 | C2O4H7NPC40H65O3 | PE 37:7e; PE 15:1e/22:6 | 1.20E+00 | 1.54E+00 | up |
| L85 | 6.23E+00 | 7.59E+02 | C2O4H7NPC41H79O3 | PE 38:1e; PE 20:0e/18:1 | 1.34E+00 | 1.07E+00 | up |
| L86 | 5.00E+00 | 7.71E+02 | C2O4H7NPC41H75O4 | PE 38:2; PE 18:1-20:1 | 1.10E+00 | 9.00E-01 | down |
| L91 | 4.71E+00 | 7.51E+02 | C2O4H7NPC41H71O3 | PE 38:5e; PE 18:1e/20:4 | 1.08E+00 | 8.92E-01 | down |
| L96 | 4.09E+00 | 7.59E+02 | C2O4H7NPC42H67O3 | PE 39:8e; PE 17:2e/22:6 | 1.03E+00 | 1.13E+00 | up |
| L102 | 6.14E+00 | 8.11E+02 | C2O4H7NPC45H83O3 | PE 42:3e; PE 18:1e/24:2 | 1.04E+00 | 1.34E+00 | up |
| L106 | 3.14E+00 | 6.93E+02 | C3H8O6PC33H63O4 | PG 30:0; PG 14:0-16:0 | 1.55E+00 | 1.47E+00 | up |
| L108 | 3.44E+00 | 7.22E+02 | C3H8O6PC35H67O4 | PG 32:0; PG 16:0-16:0 | 1.43E+00 | 1.22E+00 | up |
| L111 | 3.27E+00 | 7.48E+02 | C3H8O6PC37H69O4 | PG 34:1; PG 16:0-18:1 | 1.13E+00 | 9.32E-01 | down |
| L125 | 2.99E+00 | 8.22E+02 | C3H8O6PC43H71O4 | PG 40:6; PG 20:3-20:3 | 1.06E+00 | 7.99E-01 | down |
| L128 | 3.65E+00 | 8.54E+02 | C3H8O6PC45H79O4 | PG 42:4; PG 22:1-20:3 | 1.01E+00 | 1.16E+00 | up |
| L144 | 3.05E+00 | 8.82E+02 | C6O9H12PC41H67O4 | PI 38:6; PI 18:2-20:4 | 1.47E+00 | 1.15E+00 | up |
| L145 | 3.77E+00 | 9.16E+02 | C6O9H12PC43H77O4 | PI 40:3; PI 20:1-20:2 | 1.64E+00 | 8.39E-01 | down |
| L151 | 2.44E+00 | 5.24E+02 | C5O4H13NPC21H41O3 | LPC 18:0 | 1.32E+00 | 7.61E-01 | down |
| L154 | 4.01E+00 | 6.78E+02 | C5O4H13NPC31H59O4 | PC 28:0; PC 14:0-14:0 | 1.42E+00 | 1.12E+00 | up |
| L161 | 4.48E+00 | 7.33E+02 | C5O4H13NPC35H65O4 | PC 32:1; PC 16:0-16:1 | 1.55E+00 | 1.24E+00 | up |
| L165 | 4.76E+00 | 7.47E+02 | C5O4H13NPC36H67O4 | PC 33:1; PC 16:0-17:1 | 1.65E+00 | 8.56E-01 | down |
| L166 | 4.39E+00 | 7.45E+02 | C5O4H13NPC36H65O4 | PC 33:2; PC 16:1-17:1 | 1.07E+00 | 9.48E-01 | down |
| L170 | 6.22E+00 | 7.49E+02 | C5O4H13NPC37H73O3 | PC 34:0e; PC 18:0e/16:0 | 1.10E+00 | 1.26E+00 | up |
| L171 | 4.97E+00 | 7.61E+02 | C5O4H13NPC37H69O4 | PC 34:1; PC 16:0-18:1 | 1.36E+00 | 1.22E+00 | up |
| L172 | 5.47E+00 | 7.47E+02 | C5O4H13NPC37H71O3 | PC 34:1e; PC 17:0e/17:1 | 1.51E+00 | 1.46E+00 | up |
| L173 | 4.58E+00 | 7.59E+02 | C5O4H13NPC37H67O4 | PC 34:2; PC 16:1-18:1 | 1.39E+00 | 1.18E+00 | up |
| L175 | 4.27E+00 | 7.57E+02 | C5O4H13NPC37H65O4 | PC 34:3; PC 16:1-18:2 | 1.69E+00 | 1.19E+00 | up |
| L176 | 4.00E+00 | 7.55E+02 | C5O4H13NPC37H63O4 | PC 34:4; PC 16:1-18:3 | 1.59E+00 | 1.22E+00 | up |
| L177 | 5.45E+00 | 7.75E+02 | C5O4H13NPC38H71O4 | PC 35:1; PC 17:0-18:1 | 1.42E+00 | 7.10E-01 | down |
| L178 | 4.81E+00 | 7.73E+02 | C5O4H13NPC38H69O4 | PC 35:2; PC 17:1-18:1 | 1.23E+00 | 1.12E+00 | up |
| L179 | 4.43E+00 | 7.71E+02 | C5O4H13NPC38H67O4 | PC 35:3; PC 18:1-17:2 | 1.17E+00 | 8.48E-01 | down |
| L181 | 5.62E+00 | 7.89E+02 | C5O4H13NPC39H73O4 | PC 36:1; PC 18:0-18:1 | 1.51E+00 | 1.16E+00 | up |
| L183 | 5.06E+00 | 7.87E+02 | C5O4H13NPC39H71O4 | PC 36:2; PC 18:1-18:1 | 1.27E+00 | 1.17E+00 | up |
| L184 | 5.50E+00 | 7.73E+02 | C5O4H13NPC39H73O3 | PC 36:2e; PC 18:1e/18:1 | 1.59E+00 | 1.94E+00 | up |
| L187 | 4.38E+00 | 7.83E+02 | C5O4H13NPC39H67O4 | PC 36:4; PC 18:2-18:2 | 1.63E+00 | 1.23E+00 | up |
| L189 | 5.33E+00 | 8.01E+02 | C5O4H13NPC40H73O4 | PC 37:2; PC 17:1-20:1 | 1.60E+00 | 1.18E+00 | up |
| L191 | 6.31E+00 | 8.17E+02 | C5O4H13NPC41H77O4 | PC 38:1; PC 16:0-22:1 | 1.04E+00 | 1.17E+00 | up |
| L192 | 7.08E+00 | 8.03E+02 | C5O4H13NPC41H79O3 | PC 38:1e; PC 18:0e/20:1 | 1.54E+00 | 1.64E+00 | up |
| L194 | 6.26E+00 | 8.01E+02 | C5O4H13NPC41H77O3 | PC 38:2e; PC 18:1e/20:1 | 1.32E+00 | 1.32E+00 | up |
| L195 | 5.26E+00 | 8.13E+02 | C5O4H13NPC41H73O4 | PC 38:3; PC 20:1-18:2 | 1.29E+00 | 1.14E+00 | up |
| L196 | 4.81E+00 | 8.11E+02 | C5O4H13NPC41H71O4 | PC 38:4; PC 18:1-20:3 | 1.45E+00 | 1.07E+00 | up |
| L203 | 7.13E+00 | 8.45E+02 | C5O4H13NPC43H81O4 | PC 40:1; PC 16:0-24:1 | 1.69E+00 | 1.48E+00 | up |
| L206 | 7.02E+00 | 8.29E+02 | C5O4H13NPC43H81O3 | PC 40:2e; PC 18:1e/22:1 | 1.56E+00 | 1.44E+00 | up |
| L209 | 5.02E+00 | 8.37E+02 | C5O4H13NPC43H73O4 | PC 40:5; PC 18:1-22:4 | 1.41E+00 | 1.70E+00 | up |
| L213 | 4.39E+00 | 8.33E+02 | C5O4H13NPC43H69O4 | PC 40:7; PC 18:1-22:6 | 1.55E+00 | 1.22E+00 | up |
| L219 | 7.06E+00 | 8.71E+02 | C5O4H13NPC45H83O4 | PC 42:2; PC 18:1-24:1 | 1.68E+00 | 1.49E+00 | up |
| L221 | 6.00E+00 | 8.67E+02 | C5O4H13NPC45H79O4 | PC 42:4; PC 24:1-18:3 | 1.44E+00 | 1.21E+00 | up |
| L222 | 3.95E+00 | 8.79E+02 | C5O4H13NPC47H67O4 | PC 44:12; PC 22:6-22:6 | 1.70E+00 | 6.66E-01 | down |
| L224 | 7.29E+00 | 8.97E+02 | C5O4H13NPC47H85O4 | PC 44:3; PC 26:1-18:2 | 1.34E+00 | 7.54E-01 | down |
| L225 | 4.39E+00 | 7.04E+02 | C39H77N2O6P | SM d34:1; SM d18:1/16:0 | 1.42E+00 | 7.52E-01 | down |
| L226 | 4.85E+00 | 7.32E+02 | C41H81N2O6P | SM d36:1; SM d18:1/18:0 | 1.35E+00 | 8.03E-01 | down |
| L227 | 6.14E+00 | 8.14E+02 | C47H91N2O6P | SM d42:2; SM d18:1/24:1 | 1.61E+00 | 5.42E-01 | down |
| L228 | 6.97E+00 | 8.42E+02 | C49H95N2O6P | SM d44:2; SM d18:1/26:1 | 1.51E+00 | 6.95E-01 | down |
| L229 | 9.09E+00 | 8.53E+02 | C53H102O6 | TAG 50:0; TAG 16:0-16:0-18:0 | 1.70E+00 | 7.20E-01 | down |
| L237 | 7.53E+00 | 8.95E+02 | C57H96O6 | TAG 54:7; TAG 18:1-18:3-18:3 | 1.28E+00 | 1.46E+00 | up |
| L238 | 9.72E+00 | 9.33E+02 | C59H110O6 | TAG 56:2; TAG 16:0-16:1-24:1 | 1.51E+00 | 7.23E-01 | down |
| L239 | 9.40E+00 | 9.31E+02 | C59H108O6 | TAG 56:3; TAG 16:1-18:1-22:1 | 1.11E+00 | 7.74E-01 | down |
| L240 | 1.01E+01 | 9.61E+02 | C61H114O6 | TAG 58:2; TAG 16:0-18:1-24:1 | 1.54E+00 | 7.64E-01 | down |
| L241 | 9.79E+00 | 9.59E+02 | C61H112O6 | TAG 58:3; TAG 16:1-18:1-24:1 | 1.07E+00 | 7.95E-01 | down |
