# Supplementary material for: Grouper Interferon-Induced Transmembrane Protein 1 Inhibits Iridovirus and Nodavirus Replication by Regulating Virus Entry and Host Lipid Metabolism
Source: Front Immunol. 2021 Mar 9;12:636806. doi: 10.3389/fimmu.2021.636806 (PMC7985356; doi:10.3389/fimmu.2021.636806)
Supplement: Supplementary file 3 [file Data_Sheet_1.docx]

**
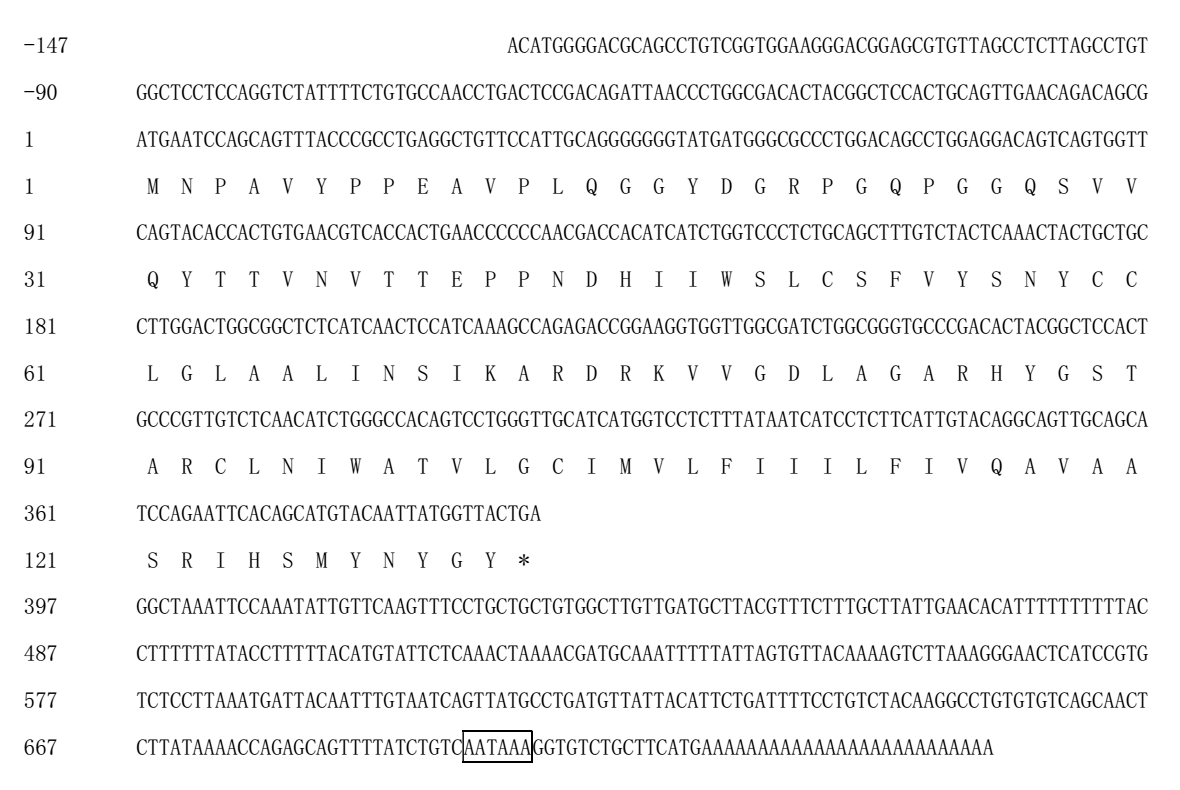
FIGURE S1 The Nucleotide sequence of EcIFITM1 and the deduced amino acid sequence.** The polyadenylation signal sequence AATAAA was labeled with rectangle.
